# Incorporation of Low Molecular Weight Chitosan in a Low-Fat Beef Burger: Assessment of Technological Quality and Oxidative Stability

**DOI:** 10.3390/foods10081959

**Published:** 2021-08-23

**Authors:** Pourya Izadi Amoli, Milad Hadidi, Zahra Hasiri, Arman Rouhafza, Aniseh Zarei Jelyani, Zahra Hadian, Amin Mousavi Khaneghah, José M. Lorenzo

**Affiliations:** 1 Department of Food Science and Technology, Science and Research Branch, Islamic Azad University, Tehran 14778-93855, Iran; Izadi.Amoli@live.com; 2 Department of Organic Chemistry, Faculty of Chemical Sciences and Technologies, University of Castilla-La Mancha, 13071 Ciudad Real, Spain; 3 College of Veterinary Medicine, Islamic Azad University of Shahrekord, Shahrekord 88137-33395, Iran; zahrahasiri7@gmail.com (Z.H.); A.rouhafza@gmail.com (A.R.); 4 Food Control Laboratory, Department of Food and Drug, Shiraz University of Medical Science, Shiraz 71348-14336, Iran; aniseh.zarei@gmail.com; 5 Department of Food Technology Research, National Nutrition and Food Technology Research Institute, Faculty of Nutrition Sciences and Food Technology, Shahid Beheshti University of Medical Sciences, Tehran 19816-19573, Iran; z_hadian@sbmu.ac.ir; 6 Department of Food Science and Nutrition, Faculty of Food Engineering, University of Campinas (UNICAMP), Campinas, São Paulo 13083-852, Brazil; 7 Centro Tecnológico de la Carne de Galicia, Rúa Galicia No 4, Parque Tecnológico de Galicia, San Cibrao das Viñas, 32900 Ourense, Spain; 8 Área de Tecnologia de los Alimentos, Facultad de Ciencias de Ourense, Universidad de Vigo, 32004 Ourense, Spain

**Keywords:** low-fat burger, chitosan, fat replacer, lipid oxidation, quality

## Abstract

In the present work, incorporating low molecular weight chitosan (LMWCH) (0, 0.5, 1, and 2%) as a fat replacer into low-fat beef burgers and technological, textural, and oxidative stability were investigated. The weight loss and shrinkage of samples decreased with the increase of LMWCH concentration. In contrast, the water-holding capacity and color of burgers were enhanced by the addition of LMWCH. The instrumental TPA results indicated an increase in the LMWCH levels, significantly increasing the hardness, springiness, and gumminess but decreasing the cohesiveness of low-fat beef burgers. The TBARS and peroxide values and free fatty acid content in the burgers supplemented with LMWCH increase slower than the control sample during refrigerated storage.

## 1. Introduction

Meat products such as burgers may be excellent sources of protein and essential nutrients in different age groups. However, several scientific pieces of evidence have shown the connection between animal fat intake and increased risk of significant health problems, including obesity, colon cancer, and cardiovascular diseases [1]. Hence, meat consumers look for healthier meat substitutes as a means to maintain good health. This new approach gives the meat industry an excellent opportunity to develop new products such as functional foods [2]. The use of fat replacer is the most efficient way to achieve low-fat products; however, the technical quality and color of meat products may be affected by the reduction of fat [3,4]. For this reason, many water retention hydrocolloids have been studied to boost gels’ development, including alginate, chitosan, gums, cellulose, and pectin [5,6].

Nowadays, demand for non-artificial food additives has increased, so natural ingredients are preferred. Chitosan is a cationic amino polysaccharide obtained by alkaline deacetylation of chitin. The application of chitosan in food and pharmaceutical products is mainly seen due to its unique bioactivities and functional properties [7]. Besides, its antimicrobial potential has other interesting characteristics, including antioxidant activity, lipid binding, water-holding capacities, and emulsifying properties [8,9]. Chitosan has illustrated considerable potential to enhance meat products’ technological and nutritional quality [10,11]. However, its utilization in the food system is limited due to its high molecular weight (50–800 kDa) and low water solubility. The molecular weight of this polysaccharide affects its biological activity [12].

Nevertheless, few studies have reported the use of chitosan as a fat replacer in meat products. This study investigates the effect of adding different concentrations of LMWCH on the technological and textural properties of low-fat beef burgers. Moreover, the oxidative stability of low-fat beef burgers during refrigerated storage was evaluated.

## 2. Materials and Methods

### 2.1. Materials

Ten prime cuts of foreshank cuts from heifer carcasses of mean age 16 months were obtained in a butcher in Gorgan, Iran, at 2 days post mortem and kept in a refrigerator (4 °C). Low molecular weight chitosan with a 50–190 kDa molecular weight and a deacetylation degree of 75–85% was provided from Sigma-Aldrich Co. (Louis, MO, USA). Spices and other additives were purchased from the local market. All the other chemicals and reagents were of analytical grade and were obtained from Sigma-Aldrich Co. (Louis, MO, USA) or Merck Chemicals Co. (Darmstadt, Germany).

### 2.2. Preparation of Low-Fat Beef Burgers

The low-fat beef burgers were produced to the following formulation: fresh beef (82%), beef back fat (4%), onion powder (4%), texturized soybean protein (2.75%), gluten (1%), burger seasoning (1%), betanin dye (beetroot red, E162) (0.025%), salt (1.5%), and water (4%). Firstly, the beef and fat were sliced and minced in an electronic mincer with a 10 mm hole plate (MG510, Kenwood, China) to make the burgers. Then, cold water and salt were added to the meat mix. Next, burger seasoning, texturized soybean protein, and the LMWCH in four levels (0, 0.5, 1, and 2% *w*/*w*) were added(Table 1) and mixed for 2 min using a blender (FP6031, Moulinex, France). The uniform dough was packed in plastic bags and transported in a freezer at −18 °C. Afterward 12 h, the dough was sliced with a hamburger slicer (CM-SL30, Changmag, Taiwan) and placed in a freezer at 75 g weight and an average diameter of 10 cm in low-density polyethylene bags. The burgers were cooked in a preheated electrical grill (SBG 106BK, Sencor, Czech Republic) at 180 °C for a total of 8 min (4 min for each side) and then heated up until the internal temperature of the geometrical center of each burger was 75 °C. The formulation of the control sample was similar to all treatments, except 10% fat was added in beef burger formulation without the addition of LMWCH (Table 1). The elaboration of burgers was replicated using the same ingredients and protocols on three different days.

### 2.3. Product Quality Analysis

#### 2.3.1. Cooking Loss

Cooking loss percentage was measured according to the method of Akwetey and Knipe [13]. A total of 20 g of beef burger samples was shaped to form a circular loop. The weight of burgers was determined before and after being cooked in an electric grill.

#### 2.3.2. Water-Holding Capacity (WHC)

The WHC of samples was determined as described by Yousefi et al. [14]. To determine the WHC, four layers of Whatman filter papers were put in 2 mL vials. The filter papers and vials were weighted before centrifugation. Then, burger samples (0.5 g) were placed into the vials. Vials were centrifuged at 14,000× *g* for 10 min. Then, the burger pieces were pulled out from the vials, and the weight of vials and filter papers was determined.

WHC (%) = (weight after centrifugation − weight before centrifugation)/(weight after centrifugation) × 100

#### 2.3.3. Shrinkage

The beef burger shrinkage was measured according to the method of Piñero et al. [15]. A difference between the uncooked burger diameter and cooked burger diameter was regarded as the percentage of shrinkage and calculated as follows:

Shrinkage (%) = (diameter of uncooked burger − diameter of cooked burger)/(diameter of uncooked burger) × 100

#### 2.3.4. Color Analysis

The surface color of the beef burger was determined with a Konica Minolta colorimeter using a three-dimensional CIE system with a 10° observer angle, 8 mm aperture, and illuminant A, and calibrated against a white tile (CR 330, Tokyo, Japan) recording L* (lightness), a* (redness), and b* (yellowness) values.

#### 2.3.5. Texture Profile Analysis (TPA)

TPA of burgers was measured in an XT-PLUS Texture Analyzer (SMS, Surrey, UK). One portion of the sample (1 × 2 cm diameter) was sliced from the center of the cooked burger, underwent a double compression cycle test of 50% compression by the cylindrical probe of 3.6 cm diameter and a cross-head speed of 2 mm/s. Five textural factors, namely, hardness (force (N) required for the first compression), springiness (distance of sample recovery after the first compression), cohesiveness (ratio of the active work completed under the second forced is placement curve to that completed under the first compression curve (dimensionless)), and chewiness (cohesiveness × springiness × hardness (cm/N)) were computed [5].

#### 2.3.6. Oxidative Stability

Lipid oxidation and pH of raw burger samples were evaluated after zero, 2, 4, 6, 8, and 10 days of storage under refrigeration (4 °C). Thiobarbituric acid reactive substances (TBARS) value was determined using the extraction method defined by Hautrive et al. [5]. Free fatty acid (FFA) and peroxide value (PV) of the burger samples were measured using a titration method according to AOAC Official Methods [16].

#### 2.3.7. pH Values

The pH of raw samples was determined using a portable Crison 507 pH-meter (Alella, Barcelona, Spain) with incorporation calibrated electrode specific for meat products.

#### 2.3.8. Statistical Analysis

All experimental data were expressed as means ± standard deviations (five treatments with three replications were used) after passing the analysis of variance (ANOVA) and Duncan’s multiple range tests using SPSS statistics software (ver. 22.0). *p* < 0.05 was accepted to be statistically significant.

## 3. Results

### 3.1. Technological Properties

Table 2 illustrates the effect of adding LMWCH at different levels on technological properties (cooking weight loss, WHC, shrinkage, and color) of low-fat beef burger samples. The weight loss and shrinkage of samples significantly decreased with the increase of LMWCH concentration. In contrast, the WHC of burgers was significantly increased by the addition of LMWCH. Chitosan demonstrates barrier capabilities to prevent water removal during the cooking process by making water molecular hydrogen bonds that make them more effective to avoid weight loss in the final product [10]. The proportion of non-meat components in the ground meat products formulations such as beef hamburgers can change the color of products. When the level of added chitosan was increased, the samples were observed to have higher lightness (L*), which is related to the moisture-retaining ability of chitosan, whereas Jo et al. [17] observed an increasing trend in L* values of pork sausages containing chitosan. Redness (a*) significantly improved with rising the concentration of LMWCH in low-fat beef burgers; this would be due to the effect of this chitosan on the burger’s texture [18]. A similar trend was reported by Sayas-Barberá et al. [19], who studied the influence of adding chitosan at different molecular weights in pork burgers. Additionally, the yellowness and redness were increased by the increment of chitosan. Jo et al. [17] confirm the yellowness results as they observed that the augmentation of chitosan increased the b* value of meat products, indicating that the chitosan’s natural color influenced the surface color of the final products.

### 3.2. Textural Properties

The impact of the increment of LMWCH on the texture profile of the low-fat beef burgers is illustrated in Table 3. The hardness of low-fat beef burgers as a maximum power needed to compress the samples increased significantly from 124 to 147.9 N when the LMWCH concentration increased from 0 to 2% (*p* < 0.05). This increase might be ascribed to the higher fat retention and lower moisture retention after the cooking process by the burgers containing LMWCH [5]. The instrumental TPA results indicated that the increment of LMWCH negatively affected the cohesiveness value (*p* < 0.05). Majzoobi et al. [20] observed that the different concentrations of carrageenan and konjac mannan increased hardness while reducing the meat-free sausages’ springiness and cohesiveness. Various authors have observed that the increment of non-meat components reduces the springiness and cohesiveness of meat products [5]. The addition of LMWCH to low-fat beef burgers caused an increase in springiness and gumminess.

Furthermore, the addition of chitosan had no significant effect on the chewiness of samples (*p* > 0.05). Estévez et al. [21] represented that the gumminess parameter relies on the hardness value, which justifies the similar trend showed by these parameters. Gumminess is the required power to disjoint a semi-solid state of the sample until swallowing.

### 3.3. Lipid Oxidation and pH during Refrigerator Storage

**Figure 1:**
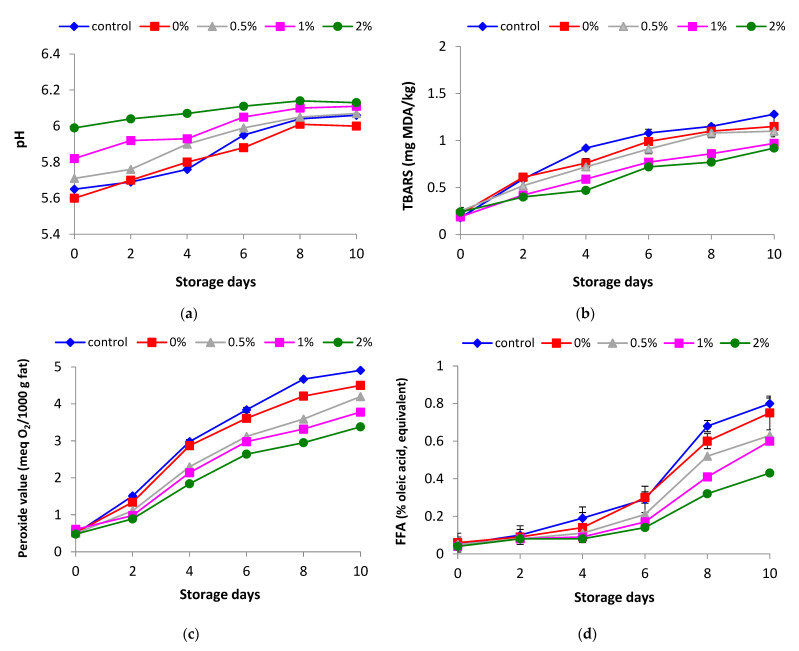
Effect of different concentrations of LMWCH (%) on pH (**a**), TBARS (**b**), peroxide (**c**), and free fatty acid (**d**) values in low-fat beef burger and control sample during 10 days of storage in the refrigerator (4 °C).

The pH values of samples varied from 5.6 to 6.13 during storage (Figure 1a). The low-fat beef burger showed higher pH than the control sample over the entire storage period. This phenomenon could be ascribed to the high pH of the chitosan used (8.75). Chitosan’s high pH can be due to its extraction method from crustacean shells, in which the chitin, a chitosan precursor, is treated with concentrated NaOH solution (40–50%) so that the reaction of deacetylation occurs, originating the chitosan [22]. Meat pH values near the myofibrillary protein isoelectric point (~5.4) reduce the WHC of myosin protein because of maximum protein–protein interaction and minimum protein–water interaction, leaking water from the meat product and negatively affecting meat quality [23,24,25]. These results agree with those reported by Turhan et al. [26] and Hautrive et al. [5], who reported higher pH values in low-fat burgers supplemented with chitosan and other fiber.

It is well known that lipid oxidation reduces meat products’ quality and sensory properties because it leads to color decrement and extension of off-flavors and off-odors [27,28]. The TBARS value of all low-fat beef burgers and control samples increased during 10 days of storage at 4 °C in the refrigerator. However, the TBARS production was significantly (*p* < 0.05) inhibited in low-fat beef burgers supplemented with chitosan, compared with the control burger over a 10-day storage period (Figure 1b). According to Dominguez et al. [29], fat contents and fatty acid composition are the key factors in the lipid oxidation of meat because fatty acids are the oxidation processes’ substrate. In this regard, the difference in TBARS values between control and 0% low-fat burgers (1.28 vs. 1.15 mg MDA/kg) could be due to the fat content since the control burgers were elaborated with 10% fat. This result agrees with data reported by other authors [30,31,32,33,34] in different meat products who observe higher lipid oxidation in meat products with high fat content. Regarding the LMWCH effect, the burger manufactured with 2% of LMWCH presented the lowest TBARS values on the last day of storage (1.1, 0.97, and 0.92 mg MDA/kg, for 0.5, 1, and 2% LMWCH, respectively). In addition, the low-fat beef burger formulated with LMWCH (0.5, 1, and 2%) at all storage days illustrated values lower than the threshold for off-flavor perception by sensory evaluation (1.0 mg MDA/kg) [35]. The result of TBARS analysis confirms the potent effect of the LMWCH in inhibiting lipid oxidation in a low-fat beef burger, such as the previously described antioxidant activity of LMWCH [5,36].

These results agree with those reported by Turhan et al. [28] and Hautrive et al. [5], who reported higher pH values in low-fat burgers supplemented with chitosan and other fiber. The TBARS value of all low-fat beef burgers and control samples increased during 10 days of storage at 4 °C in the refrigerator. However, the TBARS production was significantly (*p* < 0.05) inhibited in low-fat beef burgers supplemented with chitosan, compared with the control burger over a 10-day storage period (Figure 1b). This difference in TBARS values between control and 0% low-fat burgers could be due to the fat content since the control burgers were elaborated with 10% fat. On the other hand, the low-fat beef burger formulated with LMWCH (0.5, 1, and 2%) at all storage days indicated values lower than the threshold for off-flavor perception by sensory evaluation (1.0 mg MDA/kg) [35]. The result of TBARS analysis confirms the potent effect of the LMWCH in inhibiting lipid oxidation in a low-fat beef burger, such as the previously described antioxidant activity of LMWCH [5,36]. The results indicated an increasing trend in peroxide values for all the samples during storage, but the values were much under the maximum acceptable limit (10–20 meq/kg) for peroxide value in meat products (Figure 1c).

Further, this study indicated that the control sample’s peroxide value was significantly higher than the low-fat beef burger supplemented with chitosan at all storage days (*p* < 0.05). The replacement of fat with chitosan decreased the FFA value of beef burgers. The lowest FFA value was found in the low-fat beef burgers formulated with 2% LMWCH during storage (Figure 1d). These results agree with Jeyakumari et al. [37], who observed that the antioxidant activity of chitosan helps avoid the lipid oxidation in the Pangasius surimi supplemented by chitosan.

## 4. Conclusions

Technological, textural, and lipid oxidation of beef burgers were affected by the increment of LMWCH. The results indicated that the incorporation of LMWCH in low-fat beef burger formulations significantly enhanced the technological quality of the burgers due to their high WHC. The lipid deterioration was inhibited in a low-fat beef burger formulated with LMWCH compared with the control burger over a 10-day storage period. LMWCH successfully reduced the negative effects associated with lowering the fat content of beef burgers, which illustrated that LMWCH has more potential in acting as a proper fat replacer.

## Figures and Tables

**Table 1:** Formulations of five different beef burgers.

Sample	LMWCH (%)	Fat (%)
Control	0	10
0	0	4
0.5	0.5	4
1	1	4
2	2	4

**Table 2:** Technological properties of the control sample and low-fat beef burger supplemented with different concentrations of LMWCH (%).

Sample	Cooking Loss (%)	WHC (%)	Shrinkage (%)	Color		
				L*	a*	b*
Control	24.9 ± 2.9 ^b^	33.6 ± 3.4 ^cd^	14.4 ± 0.8 ^a^	35.4 ± 3.5 ^c^	4.8 ± 0.5 ^c^	11.1 ± 0.8 ^d^
0	27.6 ± 1.5 ^a^	32.9 ± 1.3 ^d^	13.5 ± 1.2 ^b^	33.1 ± 4.2 ^d^	4.9 ± 0.3 ^c^	12.6 ± 0.5 ^c^
0.5	21.2 ± 1.8 ^c^	34.2 ± 2.7 ^c^	13.1 ± 0.9 ^b^	35.5 ± 1.6 ^c^	5.6 ± 0.7 ^bc^	13.9 ± 0.7 ^b^
1	18.3 ± 2.3 ^d^	37.5 ± 4.1 ^b^	10.7 ± 1.5 ^c^	36.6 ± 3.8 ^b^	6.1 ± 0.3 ^b^	14.5 ± 1.1 ^ab^
2	17.1 ± 2.5 ^e^	40.1 ± 3.5 ^a^	9.8 ± 0.6 ^c^	37.9 ± 1.5 ^a^	7.6 ± 0.4 ^a^	15.2 ± 0.4 ^a^

Means ± SD in the same column with different superscripts are significantly different (*p* > 0.05). L* (lightness), a* (redness), and b* (yellowness) values.

**Table 3:** Textural characterizations of the control sample and low-fat beef burger supplemented with different concentrations of LMWCH (%).

Sample	Hardness (N)	Cohesiveness	Springiness	Gumminess	Chewiness (cm/N)
Control	124.0 ± 6.8 ^e^	0.59 ± 0.05 ^a^	5.95 ± 0.2 ^d^	59.3 ± 3.4 ^d^	450.5 ± 24.9 ^b^
0	136.3 ± 5.4 ^d^	0.51 ± 0.03 ^b^	6.26 ± 0.4 ^cd^	63.7 ± 1.9 ^c^	463.2 ± 10.6 ^a^
0.5	139.5 ± 6.0 ^c^	0.45 ± 0.01 ^c^	6.49 ± 0.6 ^c^	66.8 ± 4.5 ^b^	463.8 ± 28.2 ^a^
1	143.4 ± 4.5 ^b^	0.44 ± 0.03 ^c^	6.95 ± 0.4 ^b^	67.1 ± 2.2 ^ab^	461.3 ± 17.5 ^a^
2	147.9 ± 3.7 ^a^	0.40 ± 0.01 ^d^	7.74 ± 0.9 ^a^	68.2 ± 1.7 ^a^	462.1 ± 21.8 ^a^

Means ± SD in the same column with different superscripts are significantly different (*p* > 0.05).

## Data Availability

The data presented in this study are available on request from the corresponding author.
